# UMI‐77 Ameliorates Lipopolysaccharide‐Induced Sepsis‐Associated Encephalopathy by Modulating the Brain‐Gut Axis

**DOI:** 10.1002/brb3.71175

**Published:** 2026-01-13

**Authors:** Yu Ke, Cuicui Dong, Chang Liu, Menglu Ni, Yuting Chen, Yurou Zhang, Yingyue Wang, Yubin Xu, Guirong Chen

**Affiliations:** ^1^ College of Pharmacy Liaoning University of Traditional Chinese Medicine Shenyang China; ^2^ Department of Pharmacy Taizhou Central Hospital (Taizhou University Hospital) Taizhou China; ^3^ Department of Pharmacy Taizhou Hospital of Zhejiang Province Affiliated to Wenzhou Medical University Shenyang China

**Keywords:** brain‐gut axis, metabolomics, SAE, UMI‐77

## Abstract

**Purpose:**

Sepsis‐associated encephalopathy (SAE) is a common neurological complication of sepsis. UMI‐77 has shown unique benefits in modulating inflammation to improve sepsis. However, the exact role of UMI‐77 in the treatment of SAE and its mechanism are unknown.

**Aim:**

This article analyzes UMI‐77 based on metabolomics and explores its mechanism of action in treating SAE based on the brain‐gut axis.

**Method:**

In this study, hematoxylin and eosin (H&E) and immunofluorescence staining were used to evaluate the therapeutic effect of UMI‐77 on SAE mice. Applying untargeted metabolomics analysis, the metabolic changes in the brain and intestines of septic mice treated with UMI‐77 were examined. Furthermore, Receiver Operating Characteristic (ROC) analysis was used to select predictive biomarkers for exploring the mechanism of UMI‐77 in treating SAE.

**Findings:**

Sixty‐six significant biomarkers in the brain were found and selected with the aid of untargeted the metabolomic method. Metabolic pathway analysis indicates that these differential metabolites are mainly involved in the metabolism of linoleic acid, biosynthesis of phenylalanine, tyrosine, and tryptophan, and phenylalanine metabolism. Seventy‐eight important biomarkers were identified and selected in the intestine; metabolic pathway analysis revealed that these differential metabolites were mainly involved in phenylalanine, tyrosine, and tryptophan biosynthesis, and biotin metabolism. L‐phenylalanine, L‐tyrosine, and 5‐hydroxy‐tryptophan are the most important metabolites.

**Conclusion:**

UMI‐77 plays a positive regulatory role in disrupting the gut microbiota of mice through pathways such as the biosynthesis of phenylalanine, tyrosine, and tryptophan, and can significantly improve neurological function and reduce apoptosis of brain tissue cells.

## Graphical abstract

Metabolomics‐based analysis of SAE mice by brain‐gut axis found that metabolic pathway analysis indicates that these differential metabolites are mainly involved in the metabolism of linoleic acid, biosynthesis of phenylalanine, tyrosine, and tryptophan, and phenylalanine metabolism in the brain. Metabolic pathway analysis revealed that these differential metabolites were mainly involved in phenylalanine, tyrosine, and tryptophan biosynthesis and biotin metabolism in the intestine. Through an integrated analysis of brain and intestinal metabolomics, L‐phenylalanine, L‐tyrosine, and 5‐hydroxy‐tryptophan are the most important metabolites.

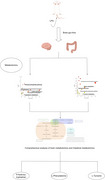

## 1 Introduction

A systemic inflammatory response syndrome brought on by infection, sepsis can lead to multiple organ dysfunction syndrome and septic shock. In the intensive care unit (ICU), it has the highest incidence and fatality (Su et al. 2012). Patients with sepsis often experience brain dysfunction; the pathogenesis of SAE is complex (Heming et al. 2017). Therefore, it is critical to investigate the pathophysiology of SAE in order to identify and create effective treatment medications.

The gut‐brain axis is a bidirectional regulatory network connecting the gastrointestinal tract and the central nervous system (CNS) through a complex network of neurological, endocrine, and immune pathways (Morais et al. 2021). Sepsis triggers a systemic inflammatory response that releases endotoxins (lipopolysaccharides) and pro‐inflammatory cytokines (IL‐6, IL‐1β, NO, and ROS). This response leads to cellular dysfunction and impaired blood‐brain barrier integrity, and neuronal degeneration and cerebral edema exacerbate neuroinflammation (Daneman and Prat 2015; Michinaga and Koyama 2015). SAE, which is also characterized by brain dysfunction, involves the gut‐brain axis (Gareau 2022). Sepsis‐induced intestinal dysfunction transmits inflammatory signals to the brain through the gut‐brain axis‐mediated neurological, endocrine, and immune pathways, thereby contributing to the development of SAE. Increasing evidence shows that the intestinal flora of SAE patients is unbalanced and the inflammatory system is abnormally activated, which leads to severe brain damage and is an important mechanism for the development of SAE (Li et al. 2021).

The U.S. Food and Drug Administration has approved UMI‐77 as a medication candidate for the treatment of cancer of the pancreas (Abulwerdi et al. 2014). Furthermore, UMI‐77, a particular medication for the treatment of Alzheimer's disease, has the ability to induce mitophagy, which results in the selective elimination of damaged mitochondria (Cen et al. 2020). We found that UMI‐77 has a significant antiseptic effect.

Previous studies have shown that 2.5 mg/kg UMI‐77 is effective in the treatment of sepsis, the mechanism of UMI‐77 in the treatment of SAE has not been reported. This study investigates the effects and mechanisms of UMI‐77 on LPS‐induced SAE mice through untargeted metabolomics analysis, providing scientific evidence for the occurrence and treatment of sepsis. It provides a research basis for further study of the biological mechanism of UMI‐77.

## 2 Materials and Methods

### 2.1 Reagent

UMI‐77 was purchased from TopScience Inc. (Shanghai, China). Lipopolysaccharide (LPS) was purchased from Sig Mardrich (Ste). Louis, Demon, Ursa).

### 2.2 SAE Model Establishment

The Taizhou University experimental animal ethics committee gave its approval to this work, which was carried out in compliance with the National Institute of Health's recommendations (ID number: TZXY‐2022‐20221015). SPF male BALB/c mice (18 ‐ 22 g) were purchased from Zhejiang Weitong Lihua Laboratory Animal Technology Co. Ltd. (Zhejiang, China; Laboratory Animal License, SCXK Zhejiang: 2021‐0013). Under SPF laboratory conditions, all animals were accommodated in temperature‐controlled groups with a 12 h light/dark cycle and free access to food and water. The experimental grouping was as follows: 24 male BALB/c mice were randomly divided into 3 groups: control group (*n* = 8), model group (*n* = 8), and UMI‐77 group (*n* = 8; 2.5 mg/kg). Mice in the model group and UMI‐77 group were given a single injection of LPS (18 mg/kg, intravenously), and the control group was given an equal volume of normal saline. Then the corresponding dose of UMI‐77 (2.5 mg/kg) was injected intraperitoneally from the UMI‐77 group once a day for 5 consecutive days. An equal volume of normal saline is given to the mice in the model and control groups. After the last dose, mice are anesthetized with sodium pentobarbital, then the mice are sacrificed, and brain and intestinal contents are obtained and stored at −80°C for further analysis. Take 3–5 brain tissues and immerse them in 4% paraformaldehyde.

### 2.3 Histological Analysis

The brain was taken, and the tissue was cut into 5 µm thick slices after ethanol dehydration, xylene cleaning, paraffin embedding, coagulation, and freezing treatment and stained with H&E. Sections were examined by making use of a light microscope (CX 33, Olympus, Tokyo, Japan) at 400x magnification. Then, the TUNEL assay kit (C1088, Beyotime, Shanghai, China) was used to label the DNA fragments of apoptotic cells according to the instructions. Apoptotic cells in brain tissue were analyzed semi‐quantitatively with the ImageJ system (V1.8.0).

### 2.4 Untargeted Metabolomics

Ultra‐performance liquid chromatography (Thermo Fisher Scientific Chemistry, Waltham, MA, USA) connected to a quadrupole off‐time device (Thermo Fisher Scientific) is used in untargeted metabolomics for observing metabolite alterations in mice treated with UMI‐77. At the same time, QC samples were tested and the method validated. Therefore, the smaller the difference in the response of the standard within the QC sample (median RSD ≤ 30%), the more stable the system and the higher the data quality. Principal Component Analysis (PCA) and Orthogonal Projection Discriminant Analysis of Latent Structures (OPLS‐DA) were performed utilizing SIMCA 14.1 software (Umetrics). SPSS 22.0 software is used for statistical analysis. Perform one‐way analysis of variance (ANOVA) on the data, and use t‐tests to compare between the two groups. Results are presented as average ± standard deviation (SD). It is thought that substantial differences are indicated by a *p*‐value < 0.05. Follow the criteria of VIP > 1.2 with a *p*‐value < 0.05 to identify potential differential metabolites. MetaboAnalyst 6.0(https://www.metaboanalyst.ca/) was adopted for metabolic pathway analysis.

## 3 Results

### 3.1 Effect of UMI‐77 on the Neuropathology and Brain Tissue Cell Apoptosis of SAE Mice

**FIGURE 1:**
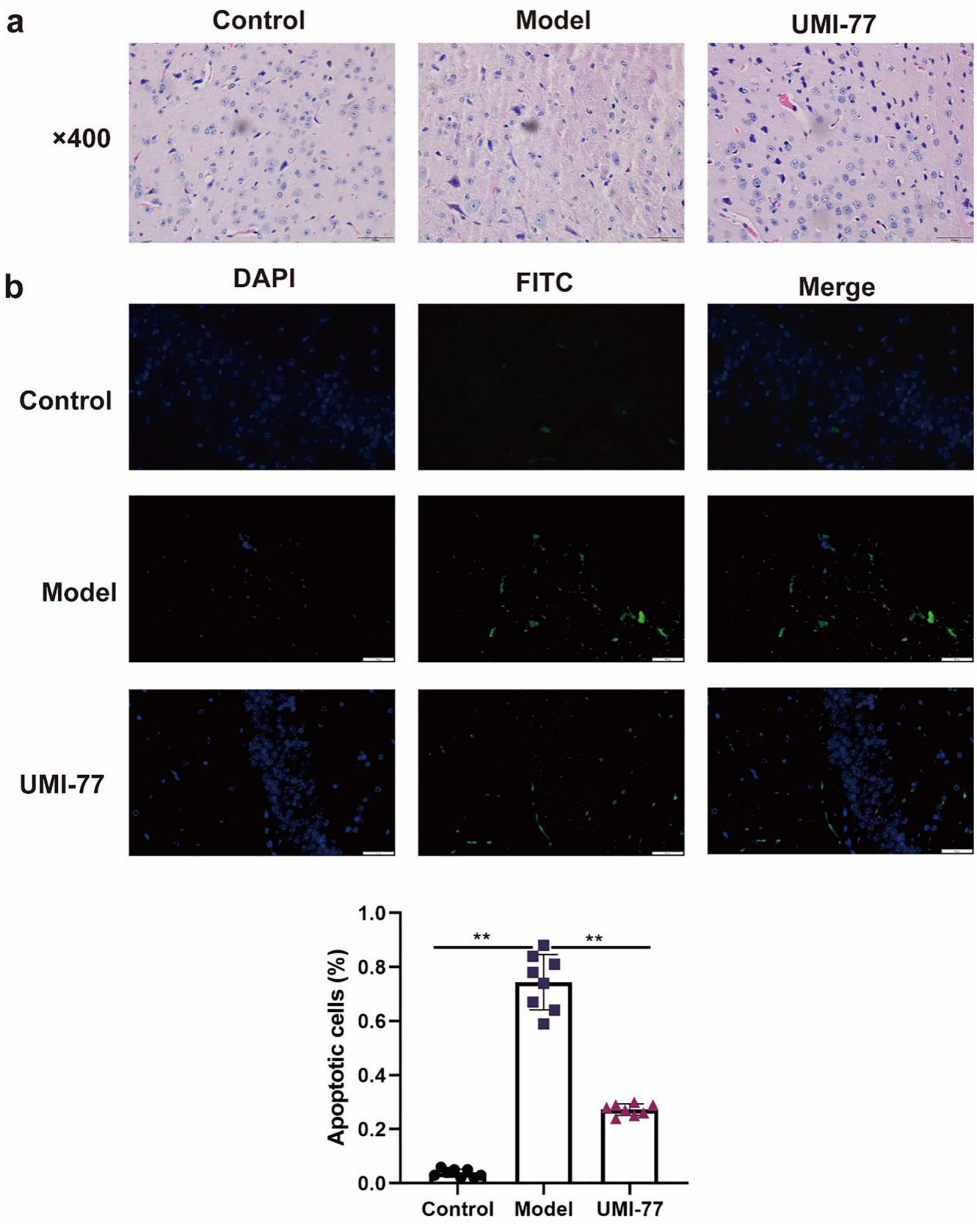
The evaluation of brain tissues. (a) H&E staining of the brain of SAE after the treatment of UMI‐77 (× 400). (b) The assessment of cellular apoptosis in brain tissues impacted by of UMI‐77 by TUNEL staining. **p < 0.01.

H&E staining (Figure 1) was applied to observe the pathological effects of UMI‐77 on septic encephalopathy in mice. The results showed that the shape and structure of glial and neuronal cells in the brain tissue of the control group of mice were clear and intact, with prominent cell body projections. The shape and structure of glial and neuronal cells in the brain tissue of the model group changed, the intercellular space increased and was ruptured, the sorting of some cells was disordered, the vascular spaces were enlarged, and congestion was observed. Compared with the model group, the degree of brain injury in the 2.5 mg/kg UMI‐77 group was significantly reduced. The impact of UMI‐77 on apoptosis in the brain tissue cells of SAE mice was examined by TUNEL staining (Figure 1).

### 3.2 Untargeted Metabolomics

**FIGURE 2:**
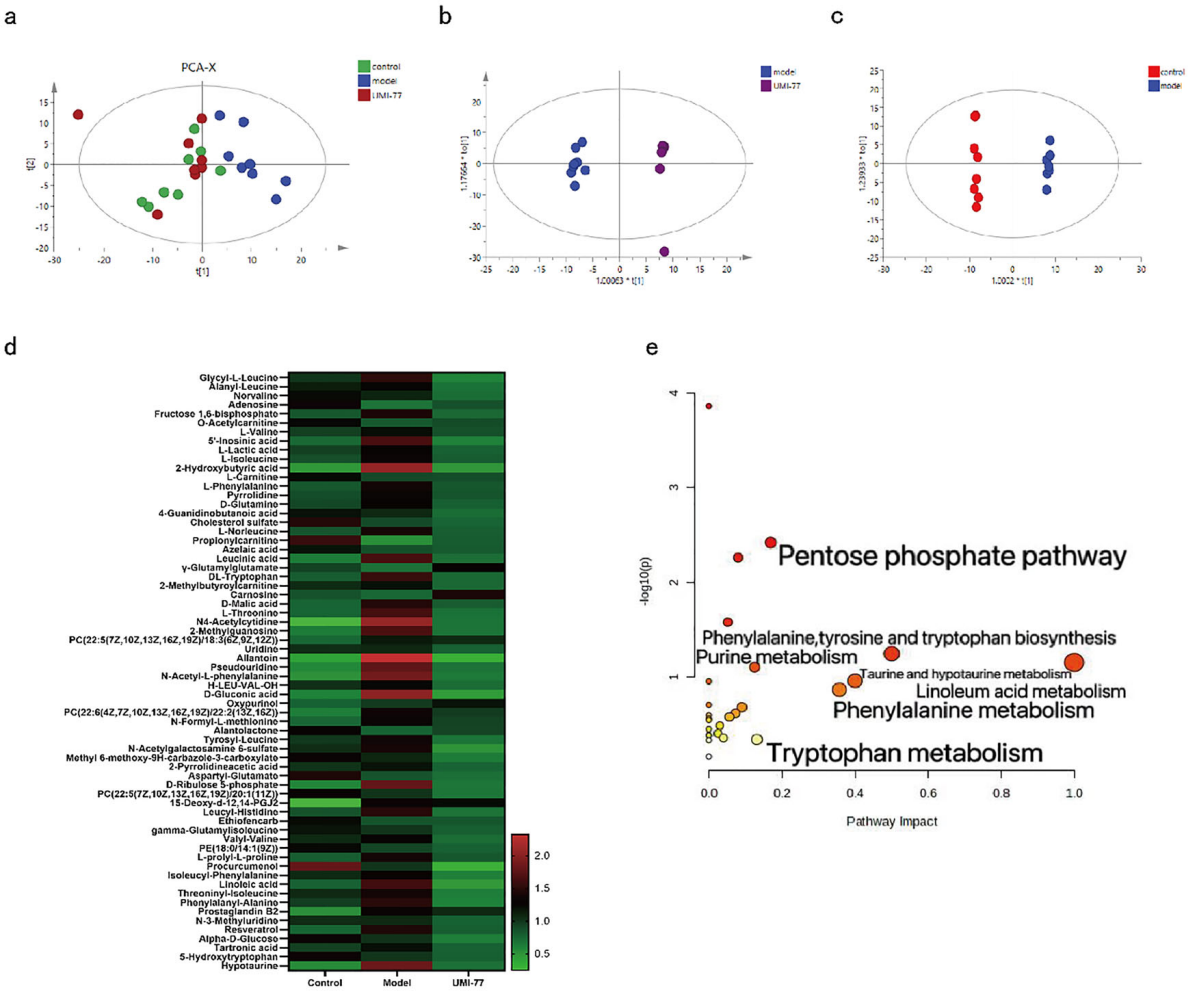
Brain tissue metabolic data of SAE mice treated by UMI‐77. (a) PCA analysis among control, model, and UMI‐77 groups. (b) OPLS‐DA analysis of control group and model group. (c) OPLS‐DA analysis of the UMI‐77 group and model group. (d) Heatmap analysis of 66 differential metabolites. (e) Pathway analysis of 66 differential metabolites.

PCA results for brain metabolites showed a significant difference between the UMI‐77 group and the model group but close to the control group (Figure 2). The variables with a *p*‐value < 0.05 and a VIP > 1.2 were identified as possible differential metabolites that could differentiate the two groups based on the OPLS‐DA model. In OPLS‐DA analysis, there was a significant separation among the control group, the UMI‐77 group, and the model group, indicating differences in metabolites (Figure 2). Based on the positive and negative ion patterns, comparing the model group to the control group, sixty‐six metabolites in total were found. At the same time, it was discovered that UMI‐77 had a reversal effect on these metabolites(Table S1). Through the analysis of the heat map of the differential metabolites mentioned above, it is evident that all 24 samples cluster into three groups (Figure 2).

The control group and UMI‐77 were clustered into one group, and the model group was clustered into another. The above indicates that UMI‐77 can improve the differences in metabolites between the model group and the control group, demonstrating therapeutic effects. The results of enrichment analysis showed that these metabolites were mainly involved in linoleic acid metabolism, phenylalanine, tyrosine and tryptophan biosynthesis, taurine and linotaurine metabolism, phenylalanine metabolism, the pentose phosphate pathway, and tryptophan metabolism (Figure 2).

**FIGURE 3:**
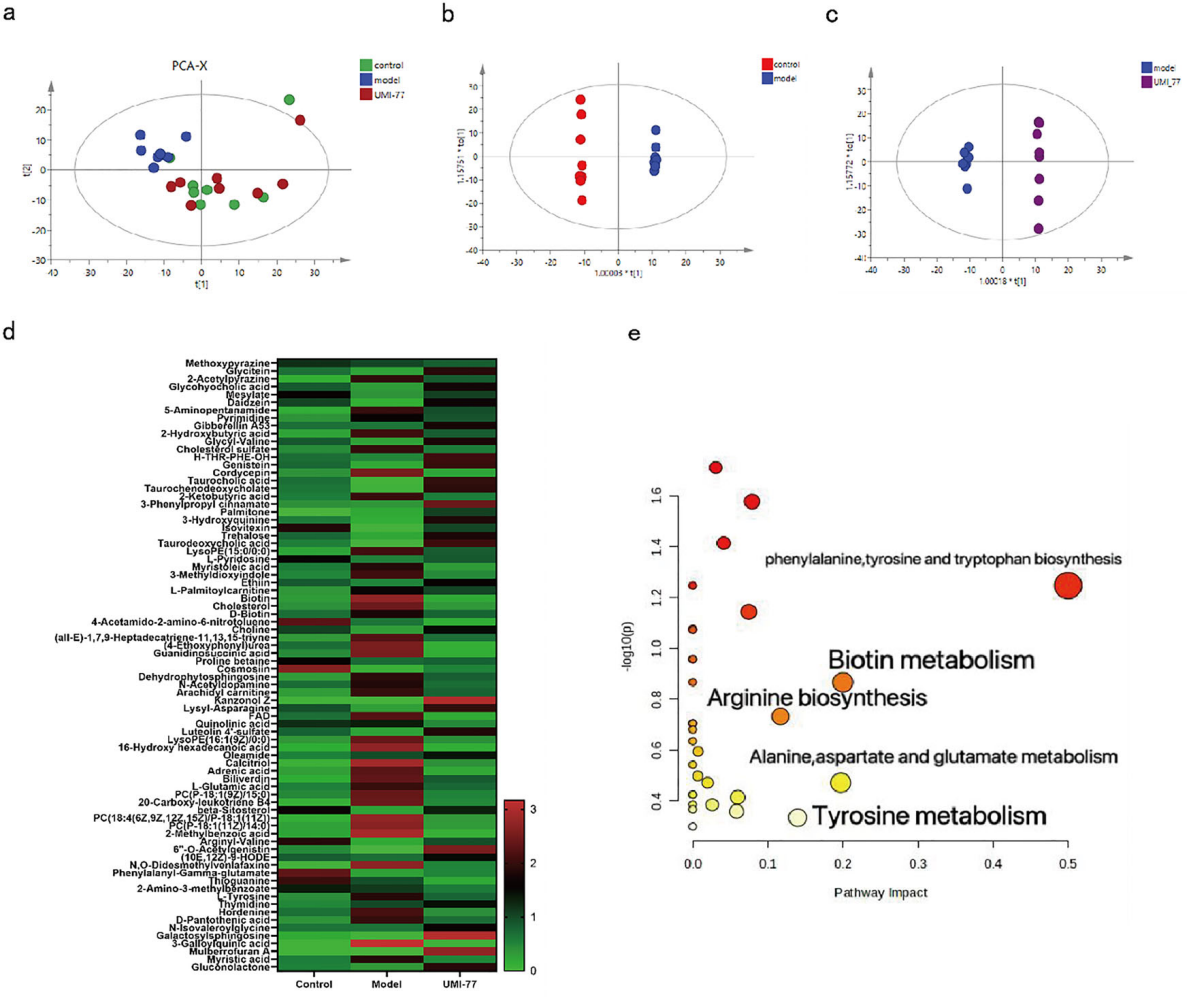
Intestinal tissues metabolic data of SAE mice treated by UMI‐77. (a) PCA analysis among control, model and UMI‐77 groups. (b) OPLS‐DA analysis of control group and model group. (c) OPLS‐DA analysis of UMI‐77 group and model group. (d) Heatmap analysis of 78 differential metabolites. (e) Pathway analysis of 78 differential metabolites.

The intestinal contents were analyzed by untargeted metabolomics, and the PCA results of intestinal metabolites were consistent with the results of brain metabolomics (Figure 3). The samples of the UMI‐77 group were significantly farther away from the model group and closer to the control group. In OPLS‐DA analysis, there was a significant separation among the control group, the UMI‐77 group, and the model group, indicating differences in metabolites (Figure 3). Based on the positive and negative ion patterns, comparing the model group to the control group, seventy‐eight metabolites in total were found(Table S2). In addition, the above metabolites were used for heatmap analysis (Figure 3), which classified the control and UMI‐77 groups into one cluster and the model group into another. The therapeutic effect of UMI‐77 on SAE mice is achieved by improving the differences in metabolites between the model and control groups. These metabolites are mainly involved in phenylalanine, tyrosine, and tryptophan biosynthesis; biotin metabolism; alanine, aspartic acid, and glutamate metabolism; tyrosine metabolism; and arginine biosynthesis (Figure 3).

### 3.3 Comprehensive Analysis of Brain Metabolomics and Intestinal Metabolomics

**FIGURE 4:**
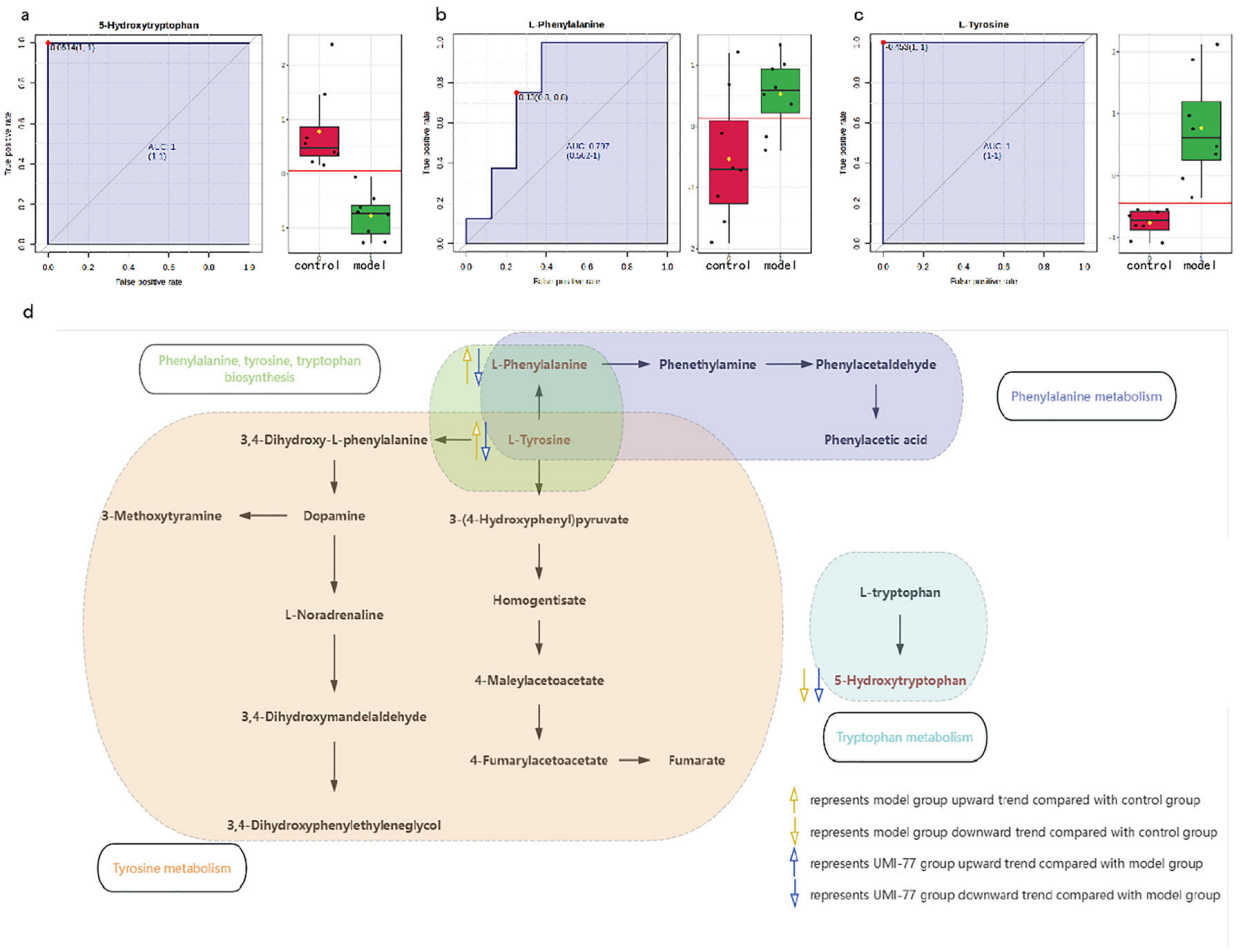
The selection and analysis of potential biomarkers. (a) ROC curve analysis of 5‐Hydroxy‐tryptophan in brain tissue with AUC = 1; (b) ROC curve analysis of L‐Phenylalanine in brain tissue with AUC = 0.797; (c) ROC curve analysis of L‐Tyrosine in intestinal tissue with AUC = 1; (d) A common pathway involved by the three metabolites.

Combining the results of brain metabolomics and intestinal metabolomics, three significant differential metabolites were obtained. They are 5‐hydroxy‐tryptophan, L‐phenylalanine, and L‐tyrosine. In this study, classic univariate ROC curve analysis was used, with the ROC curve utilized to analyze the potential biomarkers' diagnostic performance in terms of sensitivity and specificity (Wu et al. 2010). Diagnostic accuracy was analyzed by the area under the ROC curve (AUC), which was considered to have good accuracy when the AUC > 0.7 (Preston‐Martin et al. 1998). Therefore, the differential metabolites selected in the brain and gut were analyzed using MetaboAnalyst 6.0(https://www.metaboanalyst.ca/) for classical univariate ROC curve analysis. The results of classical univariate ROC curve analysis showed that the AUC of the three metabolites 5‐hydroxy‐tryptophan, L‐phenylalanine, and L‐tyrosine was greater than 0.7(Figure 4). The results showed that these metabolites were aggregated into phenylalanine, tyrosine, and tryptophan biosynthesis; phenylalanine metabolism; tyrosine metabolism; and tryptophan metabolism(Figure 4). According to previous research, some of these pathways play important roles in the pathogenesis and treatment of SAE.

## 4 Discussion

Sepsis is a complex disease involving multiple factors, including microcirculatory damage, local ischemia, cell death, release of inflammatory factors, energy metabolism disorders, and participation in the body's inflammatory response (Kuperberg and Wadgaonkar 2017). Host cells have a variety of biological functions, and metabolic analysis can reveal abnormalities in SAE tissues and cells, thus helping to discover new indicators of early diagnosis or treatment. Therefore, this study applies metabolomics based on the brain‐gut axis to explore the mechanism of action of UMI‐77 in anti‐sepsis, revealing changes in sepsis metabolites and providing references for the early diagnosis and treatment of sepsis.

In this study, it was observed that the ratio of phenylalanine to tyrosine was associated with immune activation for most aromatic amino acid‐related pathway intermediates, and effective antiretroviral therapy could reduce immune activation (Zangerle et al. 2010). Phenylalanine can compete with branched‐chain amino acids to penetrate the blood‐brain barrier. The increase in aromatic amino acids produces neurotransmitters that inhibit the central nervous system (Basler et al. 2002). Research has found that the intermediate metabolite 4‐hydroxyphenylalanine in tyrosine breakdown may inhibit the initiation and activation processes of inflammasomes, leading to the secretion of IL‐1β and reducing cell apoptosis (Wei et al. 2022). Disorders of tryptophan metabolism are implicated in the pathogenesis of SAE (Gao et al. 2016). Metabolites of the serotonin pathway were observed in this study. Pro‐inflammatory cytokines may activate indoleamine‐2,3‐dioxygenase, which depletes all tryptophan normally available for serotonin formation, thereby exacerbating cognitive impairment in sepsis (Kanova and Kohout 2021). 5‐hydroxy‐tryptophan deficiency protects mouse lungs from sepsis‐induced lung injury while reducing neutrophil extracellular trap (NET) formation in lung tissue (Huang et al. 2021).

This experiment utilized untargeted metabolomics to identify 4 key metabolic pathways in the brain and gut. Amino acid metabolism controls the activation of immune cells and the creation of antibodies, which is essential for both innate immunity and adaptive immunity (Li et al. 2007). In this study, amino acid metabolism, including phenylalanine, tyrosine, and tryptophan biosynthesis, phenylalanine metabolism, tyrosine metabolism, and tryptophan metabolism, was observed to be the most significant and widespread alteration in sepsis. Sepsis encephalopathy may be caused by metabolic disorders along amino acid levels, which are seen in sepsis patients (Basler et al. 2002). Amino acid sensing has been implicated in the control of intestinal inflammation (Ravindran et al. 2016).

In the initial stage of sepsis, a large amount of IFN‐γ is released, which can promote the activation of GTP‐cyclohydrolase 1 and increase the production of 5,6,7,8‐tetrahydrobiopterin (BH4), a cofactor for phenylalanine hydroxylase (PAH), tyrosine hydroxylase (TH), and tryptophan hydroxylase (TPH), thus affecting the metabolic pathways of phenylalanine, tyrosine, and tryptophan biosynthesis (Hu et al. 2022; Qiu et al. 2015; Strasser et al. 2017). Compared with the control group, L‐phenylalanine and L‐tyrosine were upregulated in the model group. The inflammation caused by sepsis and the level of ROS are significantly increased, causing the body to consume part of the tetrahydrobiopterin, causing phenylalanine‐tyrosine metabolism disorders (Nishijima et al. 2011). Tryptophan is metabolized by the host via the serotonin pathway as well as the kynurenine pathway. Compared with the control group, 5‐Hydroxy‐tryptophan was downregulated in the model group. Indole chemicals can be produced from tryptophan by different gut bacteria. These compounds play an important role in anti‐inflammatory effects, antimicrobial peptide secretion, and xenobiotic metabolism (Agus et al. 2018).

In summary, this study explored the molecular mechanism of UMI‐77 on SAE mice by metabolomics technology and revealed the differential metabolite pathways and metabolites in SAE mice. The basis for the mechanism of UMI‐77 in the treatment of sepsis encephalopathy is laid by this work, and future investigations should concentrate on the major targets and pathways of UMI‐77 in the treatment of SAE. UMI‐77 is in the research stage with limited clinical studies. In the future, I should conduct more in‐depth research to translate preclinical findings into future clinical applications.

## Author Contributions

**Yu Ke**: conceptualization, data curation, writing – review and editing. **Yubin Xu**: conceptualization, writing – review and editing. **Guirong Chen**: conceptualization, writing – review and editing. **Cuicui Dong**: investigation, data curation, writing – review and editing. **Yuting Chen**: investigation. **Yurou Zhang**: investigation. **Menglu Ni**: investigation, data curation. **Chang Liu**: formal analysis, writing – review and editing. **Yingyue Wang**: formal analysis.

## Funding

The study was supported by the Zhejiang Province Health Innovation Talents, the National Natural Science Foundation of China (82374008), the Taizhou Science and Technology Plan Project (21ywb36), the Zhejiang Provincial Medical and Health Science and Technology Plan Project(2024KY1823), Key Natural Science Projects of Liaoning University of Traditional Chinese Medicine, the Applied Basic Research Program of the Science and Technology Department of Liaoning Province (2022JH2/ 101300081), and the Liaoning Provincial Department of Education Reserve Project (2024‐JYTCB‐074).

## Ethics Statement

The Taizhou University experimental animal ethics committee gave its approval to this work, which was carried out in compliance with the National Institute of Health's recommendations (ID number: TZXY‐2022‐20221015).

## Conflicts of Interest

The authors declare no conflicts of interest.

## Supporting information




**Supplementary Table S1**: brb371175‐sup‐0001‐TableS1.docx

**Supplementary Table S2**: brb371175‐sup‐0002‐TableS2.docx

## Data Availability

All data in this article can be publicly shared.
