# Supplementary material for: UMI‐77 Ameliorates Lipopolysaccharide‐Induced Sepsis‐Associated Encephalopathy by Modulating the Brain‐Gut Axis
Source: Brain Behav. 2026 Jan 13;16(1):e71175. doi: 10.1002/brb3.71175 (PMC12796848; doi:10.1002/brb3.71175)
Supplement: Supplementary file 1 — Supplementary Table S1: brb371175‐sup‐0001‐TableS1.docx [file BRB3-16-e71175-s001.docx]

**Table 1 Mass data of 66 metabolites**

| Metabolite name | rtmed | mzmed | VIP | control | model | UMI-77 |
| --- | --- | --- | --- | --- | --- | --- |
| Glycyl-L-Leucine | 270.71 | 189.1231546 | 1.38 | 1.01±0.47 | 1.52±0.26*** | 0.59±0.29** |
| Alanyl-Leucine | 243.56 | 203.1388043 | 1.35 | 1.14±0.42 | 1.26±0.2*** | 0.69±0.41** |
| Norvaline | 280.32 | 118.0860321 | 1.54 | 1.23±0.27 | 1.09±0.2*** | 0.71±0.24** |
| Adenosine | 170.55 | 268.102966 | 1.22 | 1.35±0.53 | 0.68±0.4*** | 0.86±0.4** |
| Fructose 1,6-bisphosphate | 438.42 | 338.9901198 | 1.22 | 0.83±0.25 | 1.43±0.28*** | 0.74±0.35** |
| O-Acetylcarnitine | 315.84 | 204.1226385 | 1.35 | 1.24±0.3 | 0.82±0.08*** | 0.89±0.28** |
| L-Valine | 311.58 | 118.0859908 | 1.23 | 0.96±0.16 | 1.22±0.14*** | 0.87±0.16** |
| 5'-Inosinic acid | 455.56 | 347.039576 | 1.29 | 0.73±0.24 | 1.66±0.66*** | 0.6±0.3** |
| L-Lactic acid | 227.98 | 89.02428031 | 1.42 | 0.95±0.11 | 1.27±0.17*** | 0.78±0.21** |
| L-Isoleucine | 287.01 | 132.1016361 | 1.32 | 0.87±0.17 | 1.33±0.23*** | 0.81±0.23** |
| 2-Hydroxybutyric acid | 187.27 | 103.0399811 | 1.39 | 0.48±0.29 | 2.06±0.69*** | 0.49±0.18** |
| L-Carnitine | 361.45 | 220.1187252 | 1.21 | 1.22±0.28 | 0.9±0.14*** | 0.89±0.33** |
| L-Phenylalanine^a^ | 272.11 | 166.0860414 | 1.28 | 0.83±0.25 | 1.38±0.13*** | 0.84±0.24** |
| Pyrrolidine | 311.53 | 72.08064141 | 1.25 | 0.89±0.13 | 1.26±0.17*** | 0.85±0.24** |
| D-Glutamine | 447.67 | 147.0759504 | 1.22 | 0.92±0.24 | 1.3±0.18*** | 0.78±0.27** |
| 4-Guanidinobutanoic acid | 368.96 | 146.0919795 | 1.38 | 1.19±0.35 | 1.08±0.37*** | 0.72±0.23** |
| Cholesterol sulfate | 24.17 | 465.3049724 | 1.56 | 1.47±0.51 | 0.9±0.22*** | 0.76±0.29** |
| L-Norleucine | 276.44 | 132.1018324 | 1.33 | 0.84±0.23 | 1.4±0.19*** | 0.79±0.2** |
| Propionylcarnitine | 291.39 | 218.1382679 | 1.54 | 1.57±0.59 | 0.53±0.22*** | 0.81±0.3** |
| Azelaic acid | 352.37 | 187.0978958 | 1.26 | 1.18±0.42 | 0.88±0.27*** | 0.81±0.16** |
| Leucinic acid | 115.05 | 131.0712267 | 1.24 | 0.64±0.64 | 1.65±0.3*** | 0.71±0.28** |
| γ-Glutamylglutamate | 469.56 | 275.0882685 | 1.25 | 0.94±0.3 | 0.7±0.19*** | 1.3±0.62** |
| DL-Tryptophan | 275.12 | 205.096707 | 1.27 | 0.8±0.42 | 1.57±0.28*** | 0.74±0.32** |
| 2-Methylbutyroylcarnitine | 254.45 | 246.1692931 | 1.23 | 1.06±0.36 | 1.2±0.18*** | 0.74±0.25** |
| Carnosine | 431.15 | 225.0992405 | 1.46 | 0.86±0.27 | 0.74±0.24*** | 1.42±0.51** |
| D-Malic acid | 414.23 | 133.0142094 | 1.29 | 0.76±0.26 | 1.47±0.27*** | 0.8±0.39** |
| L-Threonine | 361.96 | 118.0511065 | 1.37 | 0.76±0.29 | 1.64±0.19*** | 0.69±0.36** |
| N4-Acetylcytidine | 164.36 | 284.0886312 | 1.33 | 0.25±0.31 | 2.08±0.92*** | 0.68±0.28** |
| 2-Methylguanosine | 202.69 | 298.1140508 | 1.22 | 0.64±0.16 | 1.67±0.88*** | 0.68±0.2** |
| PC (22:5(7Z,10Z,13Z,16Z,19Z)/18:3(6Z,9Z,12Z)) | 144.74 | 830.5662882 | 1.28 | 0.75±0.15 | 1.16±0.18*** | 1.07±0.33** |
| Uridine | 158.24 | 243.0623126 | 1.24 | 1.05±0.24 | 1.08±0.3*** | 0.78±0.18** |
| Allantoin | 189.09 | 157.0365759 | 1.36 | 0.41±0.4 | 2.32±1.17*** | 0.31±0.09** |
| Pseudouridine | 248.42 | 243.0622384 | 1.28 | 0.57±0.31 | 1.77±0.69*** | 0.68±0.16** |
| N-Acetyl-L-phenylalanine | 185.75 | 206.0825856 | 1.41 | 0.52±0.24 | 1.87±0.66*** | 0.64±0.2** |
| H-LEU-VAL-OH | 207.37 | 231.1698708 | 1.27 | 1.03±0.32 | 1.24±0.14*** | 0.72±0.37** |
| D-Gluconic acid | 396.96 | 195.0512331 | 1.21 | 0.59±0.24 | 2.03±1.4*** | 0.45±0.12** |
| Oxypurinol | 219.99 | 151.0263183 | 1.24 | 0.77±0.26 | 0.99±0.26*** | 1.18±0.39** |
| PC (22:6(4Z,7Z,10Z,13Z,16Z,19Z)/22:2(13Z,16Z)) | 140.31 | 886.6289828 | 1.32 | 0.63±0.36 | 1.36±0.2*** | 0.98±0.35** |
| N-Formyl-L-methionine | 202.67 | 176.038716 | 1.40 | 0.75±0.13 | 1.32±0.16*** | 0.94±0.25** |
| Alantolactone | 489.93 | 231.1350149 | 1.20 | 1.29±0.45 | 0.77±0.09*** | 0.93±0.38** |
| Tyrosyl-Leucine | 219.32 | 295.1649271 | 1.30 | 1±0.27 | 1.38±0.31*** | 0.68±0.34** |
| N-Acetylgalactosamine 6-sulfate | 181.01 | 300.0400004 | 1.31 | 1.06±0.46 | 1.41±0.61*** | 0.51±0.23** |
| Methyl 6-methoxy-9H-carbazole-3-carboxylate | 291.06 | 256.0941839 | 1.53 | 1.31±0.31 | 1.1±0.42*** | 0.64±0.31** |
| 2-Pyrrolidineacetic acid | 57.75 | 130.0856103 | 1.22 | 1.02±0.22 | 1.19±0.19*** | 0.76±0.31** |
| Aspartyl-Glutamate | 451.81 | 263.086664 | 1.29 | 1.44±0.78 | 0.85±0.22*** | 0.7±0.59** |
| D-Ribulose 5-phosphate | 448.24 | 229.0119399 | 1.29 | 0.58±0.26 | 1.8±0.69*** | 0.66±0.39** |
| PC (22:5(7Z,10Z,13Z,16Z,19Z)/20:1(11Z)) | 75.27 | 862.6231722 | 1.45 | 1.31±0.53 | 1.01±0.29*** | 0.67±0.3** |
| 15-Deoxy-d-12,14-PGJ2 | 95.92 | 315.1962823 | 1.32 | 0.25±0.34 | 1.33±0.55*** | 1.22±1.03** |
| Leucyl-Histidine | 308.45 | 269.1604834 | 1.24 | 0.86±0.33 | 1.49±0.41*** | 0.68±0.34** |
| Ethiofencarb | 398.06 | 248.0652252 | 1.36 | 1.23±0.29 | 0.85±0.18*** | 0.84±0.3** |
| gamma-Glutamylisoleucine | 425.64 | 261.144049 | 1.36 | 1.18±0.32 | 1.01±0.13*** | 0.79±0.32** |
| Valyl-Valine | 222.77 | 217.1539044 | 1.29 | 1.07±0.3 | 1.26±0.12*** | 0.72±0.41** |
| PE (18:0/14:1(9Z)) | 162.75 | 690.5044864 | 1.71 | 1.24±0.15 | 0.92±0.17*** | 0.78±0.23** |
| L-prolyl-L-proline | 424.19 | 213.1227207 | 1.23 | 0.8±0.29 | 1.4±0.24*** | 0.83±0.23** |
| Procurcumenol | 279.95 | 235.1646778 | 1.68 | 1.8±0.91 | 1.01±0.46*** | 0.3±0.17** |
| Isoleucyl-Phenylalanine | 182.70 | 279.1695412 | 1.42 | 1.07±0.37 | 1.31±0.2*** | 0.61±0.38** |
| Linoleic acid | 37.62 | 279.2331366 | 1.24 | 0.78±0.42 | 1.62±0.82*** | 0.47±0.33** |
| Threoninyl-Isoleucine | 245.36 | 233.1493824 | 1.31 | 1.07±0.42 | 1.39±0.39*** | 0.63±0.31** |
| Phenylalanyl-Alanine | 219.19 | 237.1228197 | 1.39 | 0.96±0.32 | 1.49±0.21*** | 0.6±0.35** |
| Prostaglandin B2 | 95.26 | 333.207493 | 1.20 | 0.53±0.4 | 1.3±0.49*** | 1.08±0.53** |
| N-3-Methyluridine | 158.24 | 303.0838207 | 1.23 | 1.06±0.26 | 1.07±0.33*** | 0.77±0.21** |
| Resveratrol | 96.57 | 227.0669409 | 1.35 | 0.76±0.2 | 1.45±0.2*** | 0.79±0.22** |
| Alpha-D-Glucose | 377.53 | 225.064792 | 1.72 | 1.34±0.27 | 1.02±0.22*** | 0.63±0.35** |
| Tartronic acid | 451.56 | 118.9985436 | 1.39 | 0.95±0.09 | 1.21±0.17*** | 0.78±0.16** |
| 5-Hydroxytryptophan^a^ | 198.22 | 265.0928405 | 1.50 | 1.34±0.38 | 1.01±0.21*** | 0.78±0.2** |
| Hypotaurine | 352.44 | 110.0268235 | 1.40 | 0.56±0.17 | 1.84±0.48*** | 0.7±0.32** |

**P*<0.05 compared with control group；***P*<0.05 compared with model group

^a^ confirmed with authentic standards
