# Supplementary material for: UMI‐77 Ameliorates Lipopolysaccharide‐Induced Sepsis‐Associated Encephalopathy by Modulating the Brain‐Gut Axis
Source: Brain Behav. 2026 Jan 13;16(1):e71175. doi: 10.1002/brb3.71175 (PMC12796848; doi:10.1002/brb3.71175)
Supplement: Supplementary file 2 — Supplementary Table S2: brb371175‐sup‐0002‐TableS2.docx [file BRB3-16-e71175-s002.docx]

**Table 2 Mass data of 78 metabolites**

| Metabolite name | rtmed | mzmed | VIP | control | model | UMI-77 |
| --- | --- | --- | --- | --- | --- | --- |
| Methoxypyrazine | 646.02 | 111.06 | 1.46 | 1.21±0.37 | 1.03±0.08* | 0.83±0.28** |
| Glycitein | 24.61 | 283.06 | 1.28 | 0.68±0.65 | 0.26±0.44* | 1.89±1.85** |
| 2-Acetylpyrazine | 321.72 | 123.05 | 1.38 | 0.14±0.14 | 1.95±0.8* | 0.86±1.13** |
| Glycohyocholic acid | 251.12 | 464.30 | 1.24 | 0.87±0.72 | 0.32±0.54* | 1.74±1.49** |
| Mesylate | 109.54 | 94.98 | 1.21 | 1.57±0.74 | 0.4±0.21* | 1.08±0.72** |
| Daidzein | 24.17 | 255.06 | 1.20 | 1.05±0.81 | 0.13±0.23* | 1.69±1.33** |
| 5-Aminopentanamide | 334.14 | 117.10 | 1.25 | 0.17±0.17 | 2±0.85* | 0.98±1.5** |
| Pyrimidine | 321.49 | 81.04 | 1.36 | 0.39±0.07 | 1.55±0.6* | 0.92±0.79** |
| Gibberellin A53 | 249.45 | 347.19 | 1.37 | 0.69±0.49 | 0.65±0.3* | 1.76±1.23** |
| 2-Hydroxybutyric acid | 188.73 | 103.04 | 1.24 | 0.25±0.19 | 2.07±1.39* | 0.84±1.22** |
| Glycyl-Valine | 314.78 | 175.11 | 1.27 | 0.87±0.8 | 0.27±0.09* | 1.8±1.52** |
| Cholesterol sulfate | 24.71 | 465.30 | 1.36 | 0.46±0.4 | 1.98±0.46* | 0.59±0.41** |
| H-THR-PHE-OH | 254.31 | 267.13 | 1.25 | 0.71±0.39 | 0.49±0.22* | 2.08±2.05** |
| Genistein | 23.90 | 269.04 | 1.21 | 0.76±0.61 | 0.2±0.31* | 2.02±2.36** |
| Cordycepin | 96.42 | 252.11 | 1.28 | 0.41±0.41 | 2.53±1.22* | 0.24±0.12** |
| Taurocholic acid | 212.14 | 514.28 | 1.43 | 0.7±0.53 | 0.05±0.06* | 1.97±1.45** |
| Taurochenodeoxycholate | 177.88 | 498.29 | 1.39 | 0.64±0.5 | 0.06±0.04* | 1.93±1.65** |
| 2-Ketobutyric acid | 267.97 | 101.02 | 1.21 | 0.58±0.44 | 2.02±0.9* | 0.58±0.44** |
| 3-Phenylpropyl cinnamate | 250.98 | 267.14 | 1.34 | 0.42±0.16 | 0.36±0.12* | 2.42±2.49** |
| Palmitone | 152.97 | 352.15 | 1.56 | 0.01±0.01 | 0.2±0.07* | 1.07±0.93** |
| 3-Hydroxyquinine | 386.60 | 341.18 | 1.31 | 0.61±0.72 | 0.17±0.08* | 1.83±1.83** |
| Isovitexin | 139.08 | 433.11 | 1.26 | 1.85±1.46 | 0.04±0.09* | 1.03±0.72** |
| Trehalose | 427.90 | 401.13 | 1.27 | 0.77±0.9 | 0.22±0.13* | 1.8±1.46** |
| Taurodeoxycholic acid | 143.14 | 498.29 | 1.37 | 0.55±0.5 | 0.02±0.03* | 2.06±2.02** |
| LysoPE (15:0/0:0) | 220.16 | 440.28 | 1.29 | 0.24±0.25 | 2.1±0.82* | 0.85±0.96** |
| L-Pyridosine | 469.92 | 255.13 | 1.22 | 1.54±0.94 | 0.55±0.25* | 0.88±0.4** |
| Myristoleic acid | 44.04 | 225.19 | 1.23 | 0.6±0.33 | 1.88±0.69* | 0.32±0.28** |
| 3-Methyldioxyindole | 44.76 | 164.07 | 1.35 | 0.51±0.45 | 2.09±0.67* | 0.47±0.3** |
| Ethiin | 298.15 | 166.05 | 1.26 | 0.86±0.51 | 0.51±0.24* | 1.58±0.85** |
| L-Palmitoylcarnitine | 193.47 | 400.34 | 1.26 | 0.37±0.4 | 1.64±0.84* | 1.04±0.88** |
| Biotin | 282.63 | 245.09 | 1.35 | 0.32±0.26 | 2.75±1.24* | 0.2±0.16** |
| Cholesterol | 24.17 | 369.35 | 1.35 | 0.42±0.5 | 2.44±0.75* | 0.35±0.26** |
| D-Biotin | 248.06 | 245.10 | 1.23 | 0.71±0.37 | 1.8±0.58* | 0.78±0.41** |
| 4-Acetamido-2-amino-6-nitrotoluene | 132.74 | 210.09 | 1.53 | 2.28±2.24 | 0.64±0.46* | 0.15±0.17** |
| Choline | 276.57 | 104.11 | 1.26 | 1.07±0.6 | 0.3±0.2* | 1.49±0.73** |
| (all-E)-1,7,9-Heptadecatriene-11,13,15-triyne | 347.60 | 223.14 | 1.22 | 0.34±0.38 | 2.23±1.31* | 0.68±0.82** |
| (4-Ethoxyphenyl) urea | 64.30 | 181.10 | 1.29 | 0.7±0.73 | 2.56±1.16* | 0.12±0.15** |
| Guanidinosuccinic acid | 36.15 | 176.07 | 1.25 | 0.46±0.57 | 2.54±1.15* | 0.17±0.1** |
| Proline betaine | 313.47 | 144.10 | 1.31 | 1.56±0.83 | 0.74±0.3* | 0.8±0.47** |
| Cosmosiin | 136.87 | 431.10 | 1.55 | 2.64±2.23 | 0.09±0.14* | 0.53±0.33** |
| Dehydrophytosphingosine | 173.70 | 316.28 | 1.27 | 0.31±0.34 | 1.93±1.1* | 0.83±0.63** |
| N-Acetyldopamine | 224.10 | 196.11 | 1.32 | 0.7±0.38 | 1.86±0.46* | 0.72±0.34** |
| Arachidyl carnitine | 186.94 | 456.40 | 1.46 | 0.3±0.32 | 2±0.44* | 0.79±0.61** |
| Kanzonol Z | 271.18 | 407.19 | 1.38 | 0.04±0.05 | 0.03±0.02* | 3.03±3.72** |
| Lysyl-Asparagine | 385.85 | 261.16 | 1.22 | 0.95±0.65 | 0.28±0.42* | 1.97±1.76** |
| FAD | 408.44 | 786.16 | 1.24 | 0.71±1.05 | 2.24±0.89* | 0.19±0.23** |
| Quinolinic acid | 408.49 | 166.01 | 1.24 | 1.21±0.9 | 1.37±0.47* | 0.48±0.35** |
| Luteolin 4'-sulfate | 25.41 | 367.01 | 1.22 | 0.8±0.57 | 0.26±0.47* | 1.83±1.82** |
| LysoPE(16:1(9Z)/0:0) | 219.37 | 452.28 | 1.29 | 0.36±0.41 | 2.38±0.87* | 0.43±0.41** |
| 16-Hydroxy hexadecanoic acid | 47.50 | 271.23 | 1.29 | 0.16±0.15 | 2.73±1.36* | 0.15±0.07** |
| Oleamide | 183.59 | 282.28 | 1.26 | 0.54±0.43 | 0.99±0.56* | 1.43±1.31** |
| Calcitriol | 144.70 | 399.32 | 1.41 | 0.08±0.15 | 2.91±0.79* | 0.41±0.76** |
| Adrenic acid | 36.29 | 331.26 | 1.36 | 0.29±0.38 | 2.28±0.77* | 0.2±0.07** |
| Biliverdin | 256.08 | 583.25 | 1.23 | 0.16±0.16 | 2.35±1.76* | 0.87±1.22** |
| L-Glutamic acid | 433.17 | 146.05 | 1.22 | 0.56±0.58 | 2.02±0.62* | 0.76±0.6** |
| PC(P-18:1(9Z)/15:0) | 152.78 | 730.57 | 1.24 | 0.47±0.71 | 2.38±0.82* | 0.53±0.59** |
| 20-Carboxy-leukotriene B4 | 268.38 | 365.20 | 1.34 | 0.11±0.16 | 2.42±1.28* | 0.48±0.59** |
| beta-Sitosterol | 31.35 | 397.38 | 1.26 | 1.65±0.77 | 0.26±0.13* | 1.33±0.69** |
| PC (18:4(6Z,9Z,12Z,15Z)/P-18:1(11Z)) | 146.39 | 764.56 | 1.27 | 0.12±0.17 | 2.75±1.28* | 0.34±0.34** |
| PC(P-18:1(11Z)/14:0) | 153.75 | 716.56 | 1.30 | 0.27±0.48 | 2.66±1.23* | 0.33±0.34** |
| 2-Methylbenzoic acid | 117.75 | 135.05 | 1.21 | 0.25±0.36 | 2.94±1.86* | 0.11±0.17** |
| Arginyl-Valine | 371.71 | 274.19 | 1.29 | 1.88±1.43 | 0.2±0.39* | 0.96±0.74** |
| 6''-O-Acetylgenistin | 48.97 | 475.12 | 1.20 | 0.48±0.29 | 0±0* | 2.56±3.39** |
| (10E,12Z)-9-HODE | 45.87 | 295.23 | 1.40 | 0.83±0.39 | 0.71±0.27* | 1.49±0.61** |
| N, O-Didesmethylvenlafaxine | 281.83 | 250.18 | 1.30 | 0.03±0.07 | 2.7±1.57* | 0.52±0.85** |
| Phenylalanyl-Gamma-glutamate | 394.84 | 294.14 | 1.64 | 2.33±1.71 | 0.27±0.41* | 0.5±0.55** |
| Thioguanine | 166.30 | 168.03 | 1.32 | 2.02±1.83 | 0.97±0.97* | 0.2±0.4** |
| 2-Amino-3-methylbenzoate | 288.04 | 152.07 | 1.35 | 1.36±0.69 | 1.13±0.41* | 0.6±0.27** |
| L-Tyrosine^a^ | 350.97 | 182.09 | 1.24 | 0.44±0.21 | 1.88±0.82* | 0.78±0.74** |
| Thymidine | 81.23 | 301.10 | 1.26 | 0.49±0.29 | 1.02±1.2* | 1.47±1.01** |
| Hordenine | 216.30 | 166.12 | 1.20 | 0.67±0.35 | 2.13±1.09* | 0.44±0.37** |
| D-Pantothenic acid | 280.44 | 218.10 | 1.22 | 0.39±0.48 | 1.99±1.17* | 0.72±0.6** |
| N-Isovaleroylglycine | 312.89 | 160.10 | 1.29 | 0.66±0.42 | 0.65±0.34* | 1.63±1.34** |
| Galactosylsphingosine | 240.84 | 462.34 | 1.51 | 0.18±0.16 | 0.03±0.06* | 3.07±2.97** |
| 3-Galloylquinic acid | 252.17 | 343.06 | 1.33 | 0.04±0.1 | 3.17±1.64* | 0.06±0.06** |
| Mulberrofuran A | 65.58 | 393.21 | 1.69 | 0.04±0.04 | 0.01±0.02* | 2.74±1.58** |
| Myristic acid | 41.75 | 227.20 | 1.32 | 0.58±0.32 | 1.87±0.51* | 0.51±0.19** |
| Gluconolactone | 178.43 | 177.04 | 1.32 | 0.54±0.25 | 0.36±0.26* | 1.89±1.79** |

**P*<0.05 compared with control group；***P*<0.05 compared with model group.

^a^ confirmed with authentic standard
